# Initiation of vericiguat and short-term cardiovascular function improvement in heart failure patients with and without worsened renal function

**DOI:** 10.3389/fcvm.2025.1628411

**Published:** 2025-09-01

**Authors:** Xinyu Zhang, Wei Zhou, Hui Liu, Chengquan Zhang, Yifan Qi, Xiao Yuan, Zuyi Yuan, Lizhe Sun, Jianqing She, Bowen Lou

**Affiliations:** ^1^ Cardiovascular Department, First Affiliated Hospital of Xi'an Jiaotong University, Xi'an, Shaanxi, China; ^2^ Key Laboratory of Environment and Genes Related to Diseases, Ministry of Education, Xi'an, Shaanxi, China; ^3^ Department of Medicine, Xi'an Jiaotong University, Xi'an, Shaanxi, China; ^4^ Cardiovascular Department, Wuzhong People’s Hospital, Ningxia, China; ^5^ Biobank, First Affiliated Hospital of Xi'an Jiaotong University, Xi'an, Shaanxi, China

**Keywords:** vericiguat, heart failure (HF), cardiovascular function, renal function, prognosis

## Abstract

**Background:**

Recognized as a global health issue, Heart failure (HF) is a complex clinical syndrome and often worsening due to cardiorenal interactions. vericiguat, a novel soluble guanylate cyclase (sGC) stimulator, is approved for treating heart failure (HF) with reduced ejection fraction (HFrEF). This study aims to assess the short-term improvement in cardio-function resulting from in-hospital initiation of vericiguat among patients with renal dysfunction.

**Methods:**

We conducted a single-center, retrospective cohort study encompassing 401 consecutive HF patients admitted from January 2023 to June 2024, who initiated vericiguat treatment post-admission. Following propensity score matching (PSM), the study cohort was refined to 217 patients, categorized into three groups based on renal function: 31 with severe renal dysfunction, 62 with moderate renal dysfunction, and 124 with normal renal function. All patients received oral vericiguat, with the primary endpoint being the incidence of three major cardiovascular adverse events (3P-MACE) within one year. The secondary endpoint evaluated the change in left ventricular ejection fraction (LVEF) after six months relative to baseline.

**Results:**

After six months of vericiguat therapy, a significant overall improvement in LVEF was observed (mean increase of 11.8%, *p* < 0.001). The severe renal dysfunction group demonstrated the most pronounced increase in LVEF (20.2%, *p* = 0.044), whereas the normal renal function group exhibited the most significant statistical improvement (10.9%, *p* < 0.001). Kaplan–Meier survival analysis revealed a markedly lower survival probability for the severe renal dysfunction group compared to the other groups (*p* < 0.001). The Cox proportional hazard regression model indicated a trend toward a higher risk of cardiovascular adverse events in the severe renal dysfunction group, which approached statistical significance after adjustments (HR = 137.64, *p*-value = 0.050).

**Conclusion:**

Vericiguat enhances cardiac function in HF patients, irrespective of renal function status, and merits further investigation for the optimization of treatment strategies in HF patients with renal comorbidities.

**Clinical Trial Registration:**

clinicaltrials.gov, identifier (Unique Protocol ID: 82100477-3).

## Graphical Abstract

**Figure:**
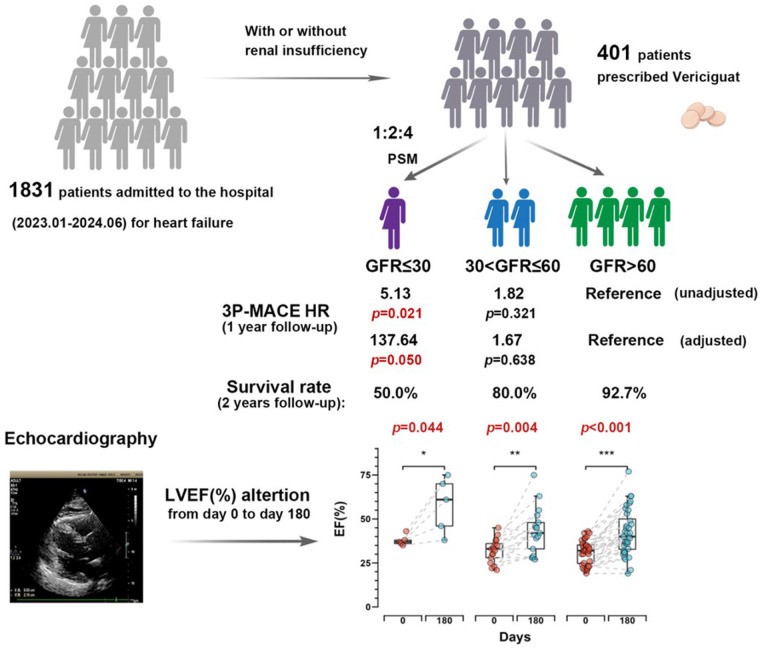
Vericiguat significantly improves cardiac function in the short term for heart failure patients regardless of renal function status. The severe renal dysfunction group showed increase in left ventricular ejection fraction (LVEF) at 20.2% (*p* = 0.044). Via both survival analysis and 3P-MACE analysis, we observed that patients with worsening renal function experience poorer outcomes within 1 year.

## Introduction

Considered as a complex clinical syndrome involving severe impairment of the pumping function, heart failure (HF) results in impairment of ventricular systolic and/or diastolic function (1). HF is recognized as a global pandemic imposing a substantial socioeconomic burden (2). According to the “Chinese guidelines for the diagnosis and treatment of heart failure 2024”, the standardized prevalence rates of HF among people aged 25–64, 65–79, and over 80 are 0.57%, 3.86%, and 7.55%, respectively (3). Patients with worsening HF events have a high risk of subsequent HF readmission and mortality.

Cardiorenal Syndrome (CRS) describes the interplay between heart and kidney, where the failure of one organ can exacerbate the damage to the other (4). In patients with heart failure, the deterioration of kidney function can further affect cardiac function, creating a vicious cycle (5). The emergence of drugs such as angiotensin receptor-neprilysin inhibitors (ARNI), beta blockers (BB), mineralocorticoid receptor antagonists (MRA), and sodium-glucose cotransporter-2 inhibitors (SGLT2i) has significantly enhanced cardiac function in patients with heart failure, earning them the designation of “new quadruple” therapy for heart failure treatment, which has been incorporated into the guidelines (6). However, the use of these agents requires monitoring of renal function and electrolytes and may be hampered by poor renal function and/or high serum potassium concentrations (7).

Vericiguat is a novel therapeutic approach that improves cardiac and renal function in heart failure patients by activating soluble guanylate cyclase (sGC) (8). Its mechanism of action gives it potential advantages in the treatment of CRS, as it can target the pathophysiological processes of both the heart and kidneys simultaneously (9). The introduction of vericiguat provides a new treatment option for heart failure patients, especially those for whom traditional treatments are limited or intolerable (10). By improving the function of both the heart and kidney, vericiguat is expected to improve the prognosis of heart failure patients, reduce the number of heart failure-related hospitalizations, and enhance the quality of life.

Therefore, the objective of this study is to assess the short-term cardiovascular function improvement in heart failure patients with and without worsening renal function (WRF), thereby providing valuable evidence for evidence-based medicine and further clinical treatment.

## Methods

### Study design and clinical data collection

This is a single-center, retrospective cohort study. 401 heart failure patients admitted consecutively from January 2023 to June 2024 at the Department of Cardiovascular Medicine, First Affiliated Hospital of Xi'an Jiaotong University, was enrolled. The inclusion criteria for patients were: (1) hospitalized due to heart failure with reduced ejection fraction (LVEF < 45%) and diagnosed with heart failure according to the Chinese Heart Failure Diagnosis and Treatment Guidelines (2024 version). (2) started treatment with vericiguat after admission. The exclusion criteria included: (1) patients with primary valvular heart disease requiring surgery or intervention, or within 3 months after valve surgery or intervention; (2) patients with rheumatic, alcoholic, or other secondary cardiomyopathies; (3) autoimmune diseases; (4) advanced cancer; (5) severe sepsis; (6) thyroid dysfunction; and (7) malignant tumors and hematological diseases. Each patient could only be included once.

In the hospital, vericiguat was administered following the Chinese consensus on heart failure management (11). The patients' medical records were collected from the Biobank of Xi'an Jiaotong University, containing identified data from the original medical records, detailed patient history, current medications, and biochemical and echocardiographic results. Follow-up information, including echocardiographic results and major cardiovascular adverse events (3P-MACE), was obtained through the Biobank and phone calls by general practitioners. Written informed consent was obtained from all study participants, and the study was approved by the Ethics Committee of Xi'an Jiaotong University First Affiliated Hospital. The study is registered at ClinicalTrials.gov (Protocol ID: 82100477-3).

### Treatment and follow-up

All patients in the cohort received oral vericiguat treatment during hospitalization and after discharge (starting dose: 2.5 mg/day, taken with food, with the dose doubling every 2 weeks, adjusted based on patient tolerance to a maximum dose of 10 mg/day). The clinical outcomes of the study included two aspects: the primary endpoint was the occurrence of three major cardiovascular adverse events (3P-MACE) within one year, including non-fatal myocardial infarction, non-fatal ischemic stroke, and cardiovascular death. The secondary endpoint was the improvement in left ventricular ejection fraction (LVEF) as measured by echocardiography after 6 months of follow-up compared to baseline.

### Propensity score matching

Due to baseline characteristic imbalances at admission, 1:2:4 propensity score matching (PSM) was used, with covariates including age, sex, and lipid profile. Patients with severe renal dysfunction (GFR ≤ 30 ml/min/1.73 m^2^) were included in the study, matched with two times the number of patients with moderate renal dysfunction (30 < GFR < 60 ml/min/1.73 m^2^) and four times the number of patients with normal renal function (GFR > 60 ml/min/1.73 m^2^). Propensity scores were calculated using R4.4.1 and the MatchIT package.

### Statistical analysis

All statistical analyses were performed using R4.4.1, SPSS 27.0 or GraphPad Prism 10. Unless otherwise stated, baseline characteristics of the three groups were expressed as the mean ± standard deviation for continuous variables, and numbers and percentages or ratios for categorical variables. One-way ANOVA was used to compare continuous variables across groups. Paired t-tests were used to compare continuous variables with normal distribution. The Chi-square test was used for categorical variables. One-way ANOVA or Fisher's exact test were used to compare continuous variables across groups. Kaplan–Meier survival curves and the LogRank test were used to estimate survival probabilities and assess statistical significance. Cox proportional hazard regression models were used to calculate the unadjusted and adjusted hazard ratios (HRs) and 95% confidence intervals (CIs). Statistical significance was defined as a two-tailed *p*-value of less than 0.05.

## Results

### Baseline characteristics of the patients

**Figure 1:**
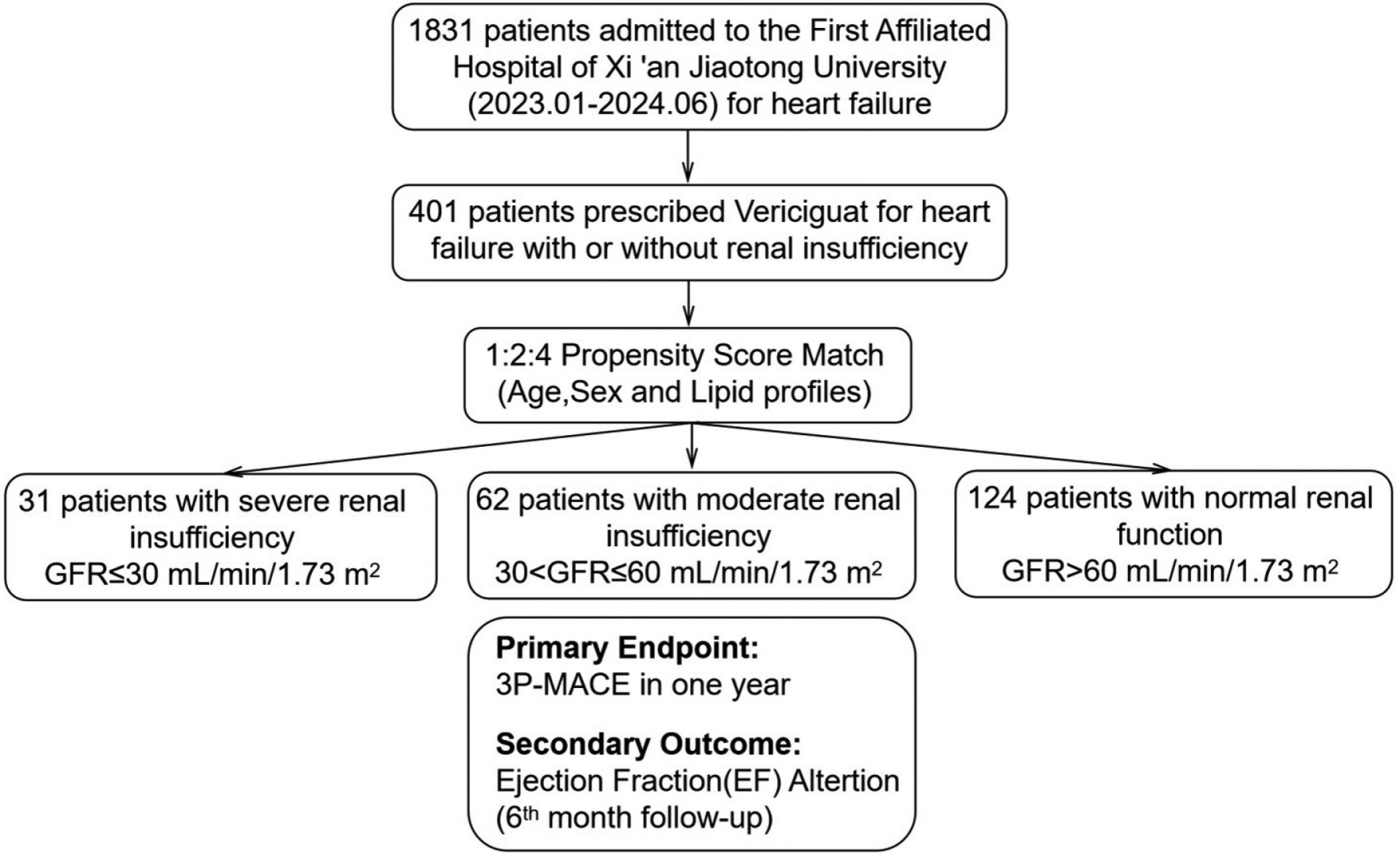
Patient selection, propensity score match and follow-up. Patients were stratified by renal function: severe renal insufficiency (GFR ≤ 30 ml/min/1.73 m^2^), moderate renal insufficiency (30 < GFR ≤ 60 ml/min/1.73 m^2^), and normal renal function (GFR > 60 ml/min/1.73 m^2^). Propensity score matching was conducted with a 1:2:4 ratio based on age, sex, and lipid profiles. The primary endpoint was 3P-MACE within one year, and the secondary outcome was Ejection Fraction(EF) alteration at 6 months.

A total of 1,831 heart failure patients admitted consecutively from January 2023 to June 2024 at the Department of Cardiovascular Medicine, First Affiliated Hospital of Xi'an Jiaotong University were screened for eligibility, 401 patients prescribed vericiguat were enrolled. After propensity score matching (PSM) based on sex, age, and lipid profile, the final cohort included 217 heart failure patients: 31 with severe renal dysfunction (GFR ≤ 30 ml/min/1.73 m^2^), 62 with moderate renal dysfunction (30 < GFR ≤ 60 ml/min/1.73 m^2^), and 124 with normal renal function (GFR > 60 ml/min/1.73 m^2^) (Figure 1). The clinical characteristics of all patients are summarized in Supplementary Table S1. As shown in Table 1, baseline characteristics were well-matched across the groups, including the use of ARNI and β-blockers, and the balance of covariates such as lipid levels. However, due to renal function constraints, complete matching could not be achieved for specific parameters. Imbalances persisted in medical history (diabetes mellitus, hypertension) and the use of MRAs/SGLT-2 inhibitors across groups, which likely stemmed from both the inherent comorbidity burden and renal function-dependent therapeutic restrictions (e.g., renal-adjusted dosing guidelines for MRAs/SGLT-2i). Furthermore, hemoglobin levels (Hgb, g/L), uric acid, and creatinine remained unmatched between groups, as these biomarkers are intrinsically modulated by renal functional status.

**Table 1:** The clinical characteristics of patients divided into three groups based on different renal functions at admission.

Variables	Total (*n* = 217)	GFR ≤ 30 (*n* = 31)	30 < GFR ≤ 60 (*n* = 62)	GFR > 60 (*n* = 124)	Statistic	*P*
Age, yrs	65.39 ± 12.02	63.13 ± 17.01	65.77 ± 11.68	65.76 ± 10.67	F = 0.64	0.530
Sex, *n* (%)						
Male	162 (74.65)	22 (70.97)	43 (69.35)	97 (78.23)	*χ*^2^ = 1.98	0.372
Female	55 (25.35)	9 (29.03)	19 (30.65)	27 (21.77)		
Smoking	85 (39.17)	8 (25.81)	25 (40.32)	52 (41.94)	χ^2^ = 2.76	0.252
Drinking	40 (18.43)	6 (19.35)	12 (19.35)	22 (17.74)	χ^2^ = 0.09	0.955
Medications						
ARNI, *n* (%)	200 (92.17)	26 (83.87)	57 (91.94)	117 (94.35)	–	0.154
MRA, *n* (%)	202 (93.09)	25 (80.65)	62 (100.00)	115 (92.74)	–	0.001***
SGLT-2i, *n* (%)	174 (80.18)	12 (38.71)	56 (90.32)	106 (85.48)	χ^2^ = 39.76	<.001***
β-Blocker, *n* (%)	200 (92.17)	27 (87.10)	59 (95.16)	114 (91.94)	–	0.368
Medical history						
Diabetes mellitus	82 (37.79)	21 (67.74)	23 (37.10)	38 (30.65)	χ^2^ = 14.54	<.001***
Hypertension	122 (56.22)	28 (90.32)	33 (53.23)	61 (49.19)	χ^2^ = 17.36	<.001***
Hgb, (g/L)	132.89 ± 21.52	114.13 ± 20.54	134.46 ± 22.18	136.85 ± 18.98	F = 15.98	<.001***
PLT, (×10^−9^)	177.77 ± 64.18	170.71 ± 57.97	170.51 ± 64.56	183.15 ± 65.42	F = 1.01	0.366
LDL, (mmol/L)	1.93 ± 0.76	1.95 ± 0.83	1.86 ± 0.78	1.96 ± 0.74	F = 0.35	0.702
HDL, (mmol/L)	0.89 ± 0.22	0.91 ± 0.25	0.85 ± 0.19	0.91 ± 0.23	F = 1.25	0.289
Hba1c, (%)	6.54 ± 1.21	6.79 ± 1.25	6.54 ± 1.03	6.48 ± 1.29	F = 0.68	0.507
Uric Acid, (mg/dl)	420.05 ± 146.42	488.45 ± 155.41	492.39 ± 140.55	366.78 ± 123.62	F = 23.07	<.001***
Cre, (*μ*mol/L)	138.21 ± 160.46	390.45 ± 307.67	128.25 ± 26.86	76.49 ± 14.15	F = 85.51	<.001***

Age, Hemoglobin (Hgb), Platelet count (PLT), Low-Density Lipoprotein (LDL), High-Density Lipoprotein (HDL), Glycated Hemoglobin (Hba1c), Uric Acid, and Creatinine (Cre) levels are presented as the mean ± standard deviation and were compared using ANOVA.

Smoking, drinking, the use of medications (ARNI, MRA, SGLT-2i, β-Blocker) and the prevalence of medical history (diabetes mellitus, hypertension) are presented as the number and percentage of patients in each category. The Chi-square test (χ^2^) was used for categorical drug data, while the Fisher exact test was applied where appropriate due to small sample sizes.

Statistical significance is indicated by asterisks: ***p < 0.001.

ARNI (Angiotensin Receptor Neprilysin Inhibitors), MRA (Mineralocorticoid Receptor Antagonists), SGLT-2i (Sodium-Glucose Cotransporter 2 inhibitors), β-Blocker (Beta-Blockers).

F: ANOVA, χ^2^: Chi-square test, -: Fisher exact.

### Cox proportional hazard regression model

The Cox proportional hazard regression model showed that in the unadjusted Model 1, the hazard ratio (HR) for the severe renal dysfunction group was 5.13 (95% CI: 1.28–20.66), with a *p*-value of 0.021, indicating a significantly higher risk of cardiovascular adverse events compared to the normal renal function group. In Model 2, adjusted for sex and age, the HR was 3.93 (95% CI: 0.25–61.26), with a *p*-value of 0.329, indicating a decrease in risk but not reaching statistical significance. In Model 3, further adjusted for baseline LVEF (%), medication history (ARNI, *β*-blockers, MRA, SGLT-2i), lipid profile (LDL, HDL), HbA1c (%), hemoglobin concentration (Hgb), and platelet count (PLT), the HR was 137.64 (95% CI: 0.99–19134.52), with a *p*-value of 0.050, approaching statistical significance (Table 2).

**Table 2:** Cox proportional hazards regression analysis used to evaluate the risk associated with different glomerular filtration rate (GFR) groups.

Variables	Model 1		Model 2		Model 3	
	HR (95% CI)	*P*	HR (95% CI)	*P*	HR (95% CI)	*P*
GFR group						
GFR > 60	1.00 (Reference)		1.00 (Reference)		1.00 (Reference)	
30 < GFR ≤ 60	1.82 (0.56–5.98)	0.321	2.46 (0.70–8.61)	0.159	1.67 (0.14–19.48)	0.683
GFR ≤ 30	5.13 (1.28–20.66)	**0** **.** **021**	3.93 (0.25–61.26)	0.329	137.64 (0.99–19134.52)	**0** **.** **050**

HR, hazard ratio; CI, confidence interval.

Bold *p*-values indicate statistical significance.

A *p*-value < 0.05 is considered statistically significant.

Model 1: Crude model with no adjustments.

Model 2: Adjusted for sex and age.

Model 3: Adjusted for sex, age, EF (%), ARNI, β-blocker, MRA, SGLT-2i, LDL (mmol/L), HDL (mmol/L), HbA1c (%), Hgb(g/L) and PLT (×10^−9^).

### Survival analysis

**Figure 2:**
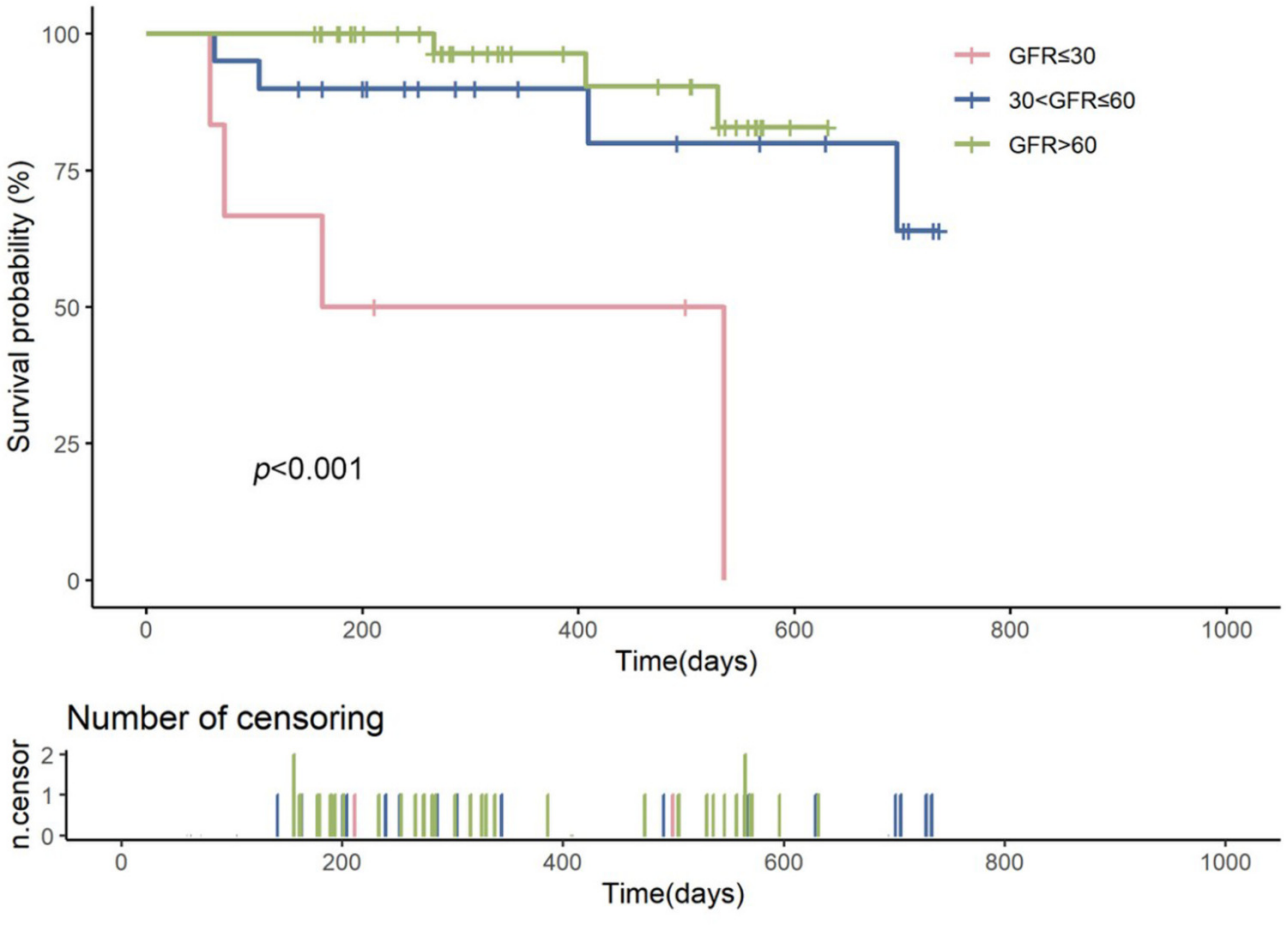
Kaplan–meier survival curves comparing the survival probabilities among patients with heart failure across different glomerular filtration rate (GFR) groups: severe renal insufficiency (GFR ≤ 30 ml/min/1.73 m^2^, shown in red), moderate renal insufficiency (30 < GFR ≤ 60 ml/min/1.73 m^2^, shown in blue), and normal renal function (GFR > 60 ml/min/1.73 m^2^, shown in green). The log-rank test *p*-value is less than 0.001, indicating a statistically significant difference in survival among the groups. The number of censored events is represented by the ticks on the time axis below the survival curves.

Kaplan–Meier survival curves showed that the survival probability for the severe renal dysfunction group was significantly lower than the other two groups, with a LogRank test result of *p* < 0.001 (Figure 2). During the follow-up, the survival rate in the severe renal dysfunction group decreased the fastest, with a survival rate of only 50.0% at 2 years. In contrast, the survival rate in the normal renal function group was the highest, with a survival rate of 92.7% at 2 years. The survival rate for the moderate renal dysfunction group was 80% at 2 years. This suggests that in heart failure patients treated with vericiguat, worsening renal function is associated with an increased risk of all-cause mortality.

### Improvement in left ventricular ejection fraction (LVEF)

**Figure 3:**
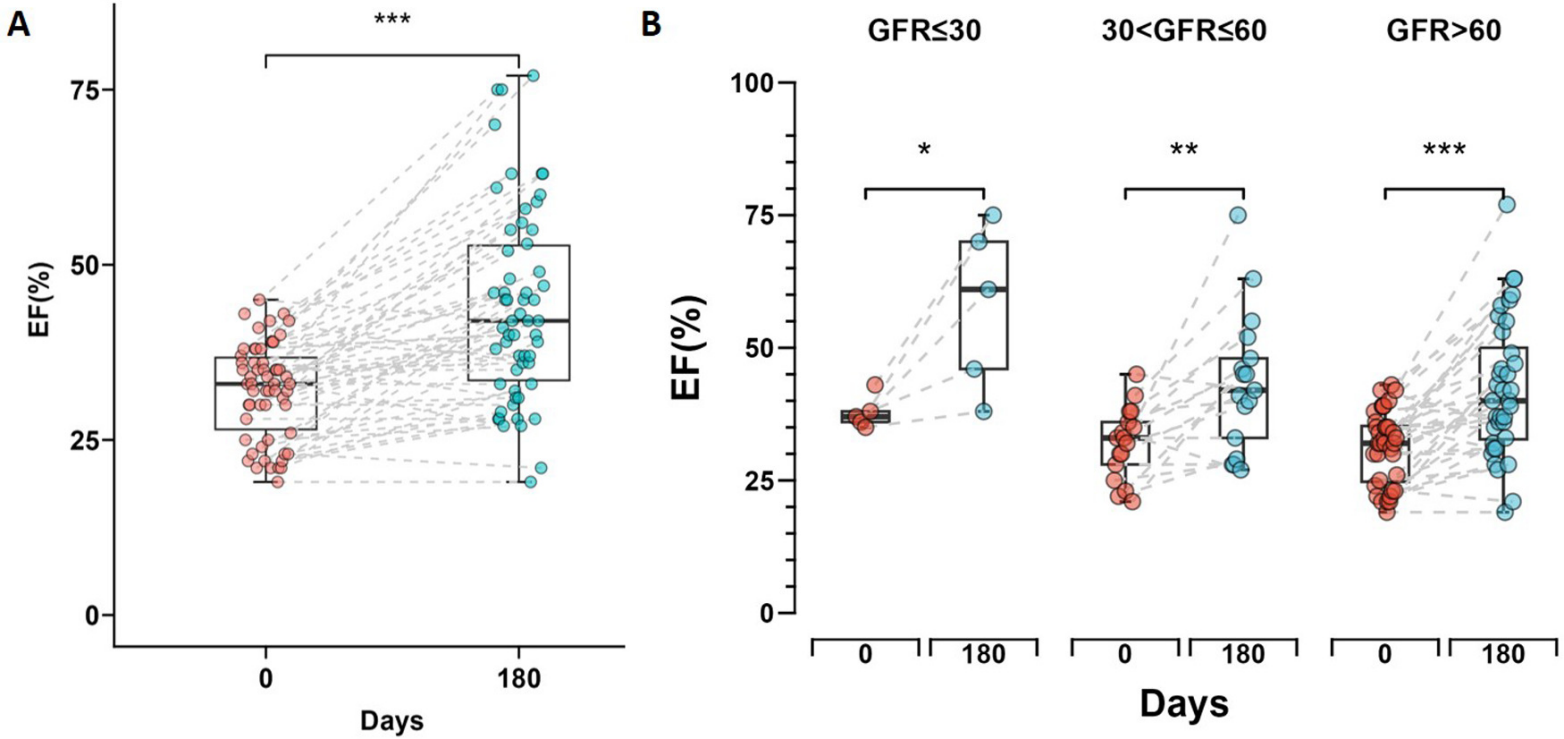
Changes in left ventricular ejection fraction (LVEF) during short-time follow-up. **(A)** Box plot showing the change in LVEF from day 0 to day 180 for all patients, with a significant increase indicated by *** (*p* < 0.001). **(B)** Box plots comparing the change in LVEF from day 0 to day 180 across three renal function groups. Statistical significance is denoted by * (*p* < 0.05), ** (*p* < 0.01) and *** (*p* < 0.001). The box plots display the median and interquartile range for each group.

After six months of treatment, the overall left ventricular ejection fraction (LVEF) of all patients measured by echocardiography showed significant improvement (*p* < 0.001) (Figure 3A), with a mean increase of 11.8%. The normal renal function group showed an overall increase in LVEF of 10.9%, with the best statistical significance (*p* < 0.001), while the severe renal dysfunction group demonstrated an LVEF increase of 20.2% which was statistically significant (*p* = 0.044). The moderate renal dysfunction group had an LVEF increase of 11.3% (*p* = 0.004). Figure 3B shows the changes in LVEF at day 0 and day 180 across the different renal function groups.

## Discussion

Through this propensity score-matched (PSM)-adjusted retrospective cohort study, we provide novel insights into the effects of vericiguat in heart failure (HF) patients stratified by renal function. Our findings demonstrate that vericiguat significantly improves cardiac function during short-term follow-up in HF patients across the spectrum of renal function, from worsening to normal. To our knowledge, this represents the first study to investigate the efficacy and clinical outcomes of vericiguat in HF patients with estimated glomerular filtration rates (eGFR) ranging from severely reduced to normal, with a focus on real-world evidence.

The interplay between heart failure and renal dysfunction, collectively termed cardiorenal syndrome and cardiovascular-kidney-metabolic syndrome (CKM), represents a major clinical challenge (12). The pathophysiology of CKM involves a multitude of factors, including inflammation, endothelial dysfunction, and hormonal imbalances, such as the renin-angiotensin-aldosterone system (RAAS) (13). These factors contribute to a vicious cycle of organ damage, where heart failure can lead to renal dysfunction, and vice versa, further complicating treatment strategies (14). Within a relatively short 6-month follow-up period, our study stands out for its timely observation of cardiac function enhancement in HF patients grappling with renal insufficiency, marked by a significant increase of approximately 11% in EF value. This early evidence of vericiguat's efficacy in bolstering cardiac function, despite the presence of compromised renal function, is a promising step forward in the quest for more effective treatments for this intricate patient population.

The pharmacological mechanism of vericiguat is particularly pertinent in CKM. By stimulating soluble guanylate cyclase (sGC) in the nitric oxide (NO)-sGC-cyclic guanosine monophosphate (cGMP) pathway, vericiguat addresses cGMP deficiency, thereby improving cardiac function and potentially exerting beneficial effects on renal function (15–17). This dual action is crucial in the management of heart failure patients with renal insufficiency, where “new quadruple” therapies (6, 18), apart from beta-blockers, have limitations when used in patients with renal impairment and hyperkalemia.

In alignment with the Victoria study (19), via both Kaplan–Meier survival analysis and 3P-MACE analysis, our findings underscore the critical observation that patients with WRF experience poorer outcomes compared to those with preserved renal function within 1 year. These WRF patients typically present with a complex array of comorbidities, which, coupled with their higher New York Heart Association (NYHA) class and elevated NT-proBNP levels, contribute to their worsened clinical outcomes (20, 21). Furthermore, renal function-dependent therapeutic restrictions (particularly for MRAs and SGLT-2 inhibitors in patients with eGFR <30 ml/min/1.73 m^2^) result in suboptimal implementation of these guideline-directed therapies. This therapeutic deficit contributes to attenuated cardiac function improvement, ultimately exacerbating adverse clinical outcomes in this high-risk population. On the other hand, the significant LVEF increases observed in the severe renal dysfunction group, despite these challenges, may reflect a particularly robust response to vericiguat in this high-risk population.

Monotherapy with vericiguat may be insufficient to completely mitigate the adverse outcomes, highlighting the need for additional research on combination therapeutic strategies. Despite advancements in HFrEF treatment, there is an unmet clinical need for novel therapies, especially for patients with both severe HFrEF and WRF. CKD patients are high risk of HF, and most patients with HF will develop some degree of CKD (22). This further emphasizes the need for therapies that can address the overlapping nature of pharmacological treatments for CKD and HF (23). The pathophysiology of HFrEF involves significant loss of cardiomyocytes and neuroendocrine activation, which plays a crucial role in the progression of renal failure (24, 25). A comprehensive understanding of these mechanisms is essential for developing effective treatments for HFrEF, and vericiguat's role in this context is promising but not exhaustive.

Our study has several methodological strengths, including the demonstration of vericiguat's beneficial effects on cardiac function in patients with a wide range of renal function (from severely impaired to normal eGFR) during the 6-month follow-up. While randomized controlled trials (RCTs) remain the gold standard for establishing efficacy under controlled conditions, their strict inclusion/exclusion criteria often limit generalizability to real-world populations, particularly those with multimorbidity, polypharmacy, or advanced renal dysfunction-groups frequently underrepresented in RCTs. This real-world evidence (RWE) complements RCT findings by validating the drug's effectiveness in a broader, unselected cohort, thereby enhancing the external validity of prior trial results. Our study, despite its retrospective design, employed rigorous propensity score matching to minimize confounding and replicate a quasi-experimental framework, thereby strengthening causal inference. The observed LVEF improvements, even in the severe renal dysfunction group, suggest that vericiguat's benefits extend beyond the idealized conditions of RCTs, supporting its utility in routine care where comorbidities like chronic kidney disease (CKD) are prevalent.

Admittedly, our study has limitations inherent to its single-center, retrospective design. The reduced sample size, particularly in the severe renal dysfunction cohort post-PSM (GFR ≤30 ml/min/1.73 m^2^, *n* = 31), though statistically powered to detect LVEF changes, may limit subgroup analyses and potentially affect the stability of the HR estimates. However, this reflects the real-world challenge of enrolling patients with advanced CKD, further underscoring the need for multicenter collaborations to aggregate larger datasets. Additionally, the 6-month follow-up, while sufficient to capture short-term cardiac functional changes, precludes assessment of long-term renal outcomes or hard endpoints like mortality. Future studies should integrate serial renal function assessments to evaluate vericiguat's bidirectional cardiorenal effects and incorporate extended follow-ups. This would determine the durability of benefits and confirm these findings in larger cohorts, thus strengthening the evidence base and ensuring result reliability regarding vericiguat's impact on patients with renal dysfunction.

## Conclusion

Through this retrospective, PSM-adjusted cohort study, our findings underscore the potential of vericiguat as a valuable addition to the therapeutic arsenal for HF patients, particularly those with comorbid renal dysfunction, and highlight the need for continued research to optimize treatment strategies in this challenging patient population.

## Data Availability

The data that support the findings of this study are not publicly available due to containing information that could compromise the privacy of research participants but are available from the corresponding author upon reasonable request. Requests to access the datasets should be directed to Bowen Lou, bowenlou@foxmail.com.
