# Supplementary material for: Initiation of vericiguat and short-term cardiovascular function improvement in heart failure patients with and without worsened renal function
Source: Front Cardiovasc Med. 2025 Sep 1;12:1628411. doi: 10.3389/fcvm.2025.1628411 (PMC12433936; doi:10.3389/fcvm.2025.1628411)
Supplement: Supplementary file 1 [file Table1.docx]

| **Variable** | **Value** |
| --- | --- |
| Age, yrs | 65.39 ± 12.02 |
| Sex, M/F number | 162/55 |
| Drugs |  |
| ARNI, n(%) | 200 (92.17) |
| MRA, n(%) | 202 (93.09) |
| SGLT-2i, n(%) | 174 (80.18) |
| β-Blocker, n(%) | 200 (92.17) |
| Hgb(g/L) | 132.89 ± 21.52 |
| PLT(×10^9) | 177.77 ± 64.18 |
| LDL(mmol/L) | 1.93 ± 0.76 |
| HDL(mmol/L) | 0.89 ± 0.22 |
| HbA1c(%) | 6.54 ± 1.21 |
| Uric Acid(mg/dl) | 420.05 ± 146.42 |
| Cre(μmol/L) | 138.21 ± 160.46 |

Supplementary Table 1 | The clinical characteristics of all patients. Baseline characteristics of all patients were expressed as the mean ± standard deviation for continuous variables, or numbers and percentages or ratios for categorical variables.
